# Novel insights into MSC-EVs therapy for immune diseases

**DOI:** 10.1186/s40364-019-0156-0

**Published:** 2019-03-18

**Authors:** Peilong Lai, Jianyu Weng, Liyan Guo, Xiaomei Chen, Xin Du

**Affiliations:** 1 Department of Hematology, Guangdong Provincial People’s Hospital, Guangdong Academy of Medical Sciences, Guangzhou, Guangdong 510080 People’s Republic of China; 2 Guangdong Geriatrics Institute, Guangzhou, Guangdong 510080 People’s Republic of China

**Keywords:** Mesenchymal stromal cells, Extracellular vesicles, Immunomodulation, Graft-versus-host disease, Inflammatory diseases

## Abstract

Mesenchymal stromal cells (MSC) are a heterogeneous cell population with self-renewal and the ability to differentiate into different lineages. The novel regulatory role of MSC in both adaptive and innate immune responses got extensive investigation and MSC have been widely used in clinical trials as immunosuppressive agents for autoimmune and inflammatory diseases, including graft-versus-host disease (GVHD), multiple sclerosis (MS), systemic lupus erythematosus (SLE), chronic kidney disease, etc. Recent studies have found that MSC exerted their immunomodulation function through secreting extracellular vesicles (EVs), which delivered parent cell cargo to recipient cells without oncogenicity or variability. Since MSC-EVs exhibit most of the properties of MSC and take advantage of their cellular immunomodulatory fuction, MSC-EVs appear to a promising none-cell therapy in various human diseases. In this review, we summarize the pivotal roles of MSC-EVs as agents for immunotherapy in diseases.

## Background

Mesenchymal stromal cells (MSC) are a type of non-hematopoietic pluripotent stem cells with self-renewal and the ability to differentiate into mesodermal tissues, such as adipogenic, chondrogenic, and osteogenic cells [1]. Emerging evidence has shown that MSC play an immunomodulatory and homeostatic role, and the application of MSC appears to offer a strategy for regulating inflammation and promoting the rehabilitation of the wounded tissue in inflammatory diseases [2]. To date, over 700 clinical trials have been registered with ClinicalTrials.gov worldwide for the use of MSC as immunosuppressive agents for autoimmune diseases or tissue regeneration therapy, including graft-versus-host disease (GVHD), multiple sclerosis (MS), systemic lupus erythematosus (SLE), Crohn’s disease, diabetes mellitus, and organ transplantation.

As early as 2004, the first human study of the immunoregulation of MSC began with GVHD. A patient with steroid-resistent, ciclosporin-resistent, grade IV acute GVHD of the gut and liver received bone-marrow-derived MSC (BM-MSC) infusion, which induced a striking clinical response and ameliorated the GVHD symptoms, particularly gut GVHD [3]. Subsequently, a phase II clinical study in which MSC were administered to 55 patients with severe steroid-resistant GVHD demonstrated an almost 70% overall response rate [4]. Although most clinical trials and animal disease models have demonstrated the efficacy of MSC transplantation in treating GVHD and other diseases, some clinical results have provided evidence of conflicting effects of MSC therapy [5]. The results of a randomized controlled, double blind phase III clinical study on MSC therapy for hormone-resistant aGVHD demonstrated that there was no difference between MSC and control groups, but the MSC had a therapeutic response to partial liver and gastrointestinal GVHD and had a strong therapeutic response to an affected child (86% vs. 57%, *p* = 0.094, *n* = 28), but no statistically significant difference was found. Similarly, in a phase III clinical study of Crohn’s disease, which was discontinued in 2009, three cases had MSC therapeutic responses, and three cases were aggravated and required surgical intervention [5]. Although many possible reasons exist for the inconsistent outcomes of these trials, it is possible that variations in the in situ inflammation status of recipients differentially affect the activation status of MSC. The variability in the efficacy in clinical studies of MSC is influenced by many factors, including source of MSC, culture system, dosage, mode of administration, research design, and evaluation criteria, and the most likely reason for this variability is that the different stages of disease and variations in the in situ inflammation status of recipients differentially affect the activation status of MSC [6, 7]. Therefore, it is important to prevent MSC from receiving environmental regulation and causing immune polarization in the inflammatory microenvironment.

In recent years, it has been reported that MSC exhibit immunomodulatory function through cell-to-cell contact and/or a paracrine pathway with the latter prominent. Many reports have showed that MSC perpetuate immunosuppressive signaling via secreted paracrine mediators rather than via cell-to-cell contact [8, 9]. The immunomodulatory therapeutic benefits of MSC administration are mainly attributed to the paracrine effects mediated by MSC-derived extracellular vesicles (MSC-EVs) [10–12]. EVs are heterogeneous vesicles bound by a phospholipid bilayer that function as mediators of intercellular communication via their loaded proteins, RNA, and/or DNA. EVs derived from MSC could exhibit most of the properties of MSC and take advantage of their cellular immunomodulatory fuction. It is worth noting that MSC-EVs have been reported to have therapeutic effects on the treatment of GVHD and chronic kidney disease in the clinic, and a variety of pre-clinical of immune disease models indicate that MSC-EVs are effective (See Table 1). In this review, we summarize current knowledge of the therapeutic potential of MSC-EVs in immune diseases.

**Table 1:** MSC-EVs as a potential therapy invarious disease models

Disease model	Potential molecular mechanism	Effect on immune cells	Refs
Type 1 diabetes	increase the levels of TGF-β, IL-10, IL-6, and PGE2 cytokine	strengthen Treg function	[45, 63, 64]
Multiple sclerosis	increase the levels ofTGF-β, IL-10, PD-L1, and galectin-1	inhibitauto-reactive lymphocyte proliferation; promote generation of Tregs	[44]
Systemic Lupus Erythematosus	transfer the Fas receptor to reduce the intracellular miR-29b levels	rescue bone marrow MSC function	[55]
Uveitis	decrease levels of IL-2 and IFN-γ	inhibit activation of APCs and the development of Th1 and Th17 cells	[56]
Osteoarthritis	increase the secretion of IL-10 Bregs	decrease the plasmablast population	[57–59]
GVHD	decrease the levels of IL-1β, TNF-α, and IFN-γ; increase the levels of IL-10 and TGF-β	reduce CD3 + CD8+ T cell and Th17 cells; induce Treg	[60–62]
Kidney injury	increase the secretion of IL-10	reduce recruitment of macrophages	[66, 68]
Cutaneous wound	shuttle let-7b	promote M2 macrophage activation	[34, 71]

### The characteristics and cargoes of MSC-derived EVs

EVs exert functions mainly depending on their parent cells. When released into the extracellular microenvironment, EVs play an indispensable role in transferring bioactive cargo, such as functional microRNAs (miRNAs), messenger RNAs (mRNAs), proteins including soluble factors, and mitochondria, thereby triggering specific intracellular signaling pathways and influencing cell fate [13–16]. EVs are generally classified into three major types according to the size, intracellular origin, and specific membrane markers. The first type is exosomes, which are vesicles 40–150 nm in size that are derived from the inward budding of multivesicular bodies (MVBs) [17–19]. Larger one is microvesicles, which range from 100 to 1000 nm, are directly released by budding and fission from the plasma membrane [20, 21]. Another one is apoptotic bodies, the largest of the EVs, ranging up to 5000 nm, form as a cell begins to degrade during the late stage of apoptosis and released by direct budding of the membrane [22, 23]. Nearly all the cells in various biological fluids can release EVs including urine, plasma, saliva, cerebrospinal fluid, synovial fluid, and breast milk under physiological or pathological conditions [24]. Because of their overlapping biophysical characteristics, coexistence in vitro and in vivo, and the lack of discriminating markers, the International Society of Extracellular Vesicles (ISEV) recommended that the term EV be used to mainly describe microvesicles and exosomes, though the latter are most abundant [25].

All EVs have surface molecules that target recipient cells. Once taken up by target cells, EVs can induce signaling via three main mechanisms: i) internalization by endocytic compartments, ii) direct fusion with membranes, and iii) recognition of specifc receptors and induction intracellular signaling pathways [26]. During cell-to-cell communication, recipient cells utilize MSC-EVs to alter their destiny. Among the complicated bioactive cargoes, microRNAs, which are noncoding RNAs that mediate RNA silencing and posttranscriptional gene repression, are the most important and extensive genetic messengers that have generated the most extensive research interest [27, 28]. miRNA species shuttled by EVs and maintaining their function have been reported in the intercellular regulation of immune modulation [29–31]. One study has demonstrated that MSC-EVs express miR-21 to promote TGF-β signaling. In support of this study, another report further showed that depletion of RNA in MSC-EVs reduce TGF-β transcripts in PBMCs [32, 33]. Indeed, Ti’s team demonstrated that EVs secreted by LPS-treated UC-MSC are anti-inflammatory and immunomodulatory, and these effects are at least partially mediated by miRNA cargo in EVs via delivery of the miRNA let-7b in LPS-pretreated UC-MSC-EVs [34]. Furthermore, Lin’s team confirmed that MSC can transfer EV-derived miR-124 to astrocytes, enhancing their anti-inflammatory effects and benefitting neurite remodeling and functional recovery by increasing the expression of glutamate transporters [35]. Therefore, it is not surprising that miRNAs are partially transferred by MSC-EVs as cargo during the resolution of inflammation. This scenario would be an attractive therapeutic avenue because MSC-EVs, by transferring mRNA or miRNA, can induce protein expression in target cells.

Proteomic analyses have indicated that MSC-EVs are composed of two broad classes of proteins: cell membrane and cytoplasmatic proteins associated with intracellular vesicle biogenesis and trafficking and proteins associated with self-renewal and differentiation and soluble mediator proteins [36]. In immune-related diseases, soluble factors encased in EVs play an important role in maintaining tissue homeostasis and regulating immune balance, such as IL-10, TGF-β, INF-γ, IDO, and prostaglandin E2, which can effective induce monocyte transformation into regulatory T cells [37, 38]. Treatment of EV-producing cells with specific growth factors can change the phenotype and protein content of EVs and increase the effectiveness of EVs. A study reported that EVs derived from MSC overexpressing pigment epithelium-derived factor (PEDF), a multifunctional protein that exhibits anti-inflammatory, antioxidative, and neuroprotective properties, ameliorated cerebral ischemia-reperfusion injury more than that for a control group, because the increase in PEDF content in EVs promoted protective effects against the oxygen-glucose deprivation-induced apoptosis by activating autophagy [39]. In addition to carrying miRNA and proteins, MSC-EVs can also transfer mitochondria, which demonstratates that they are capable of racapitulating the beneficial therapeutic effects of MSC. In a model of silicosis, a study reported that MSC transfer miRNA and mitochondria via EVs to human macrophages, which suppresses macrophage Toll-like receptor signaling by miRNA but enhances macrophage bioenergetics by mitochondria [11]. Another study indicated that MSC-EVs can mediated mitochondrial transfer to macrophages, which induce an anti-inflammatory and highly phagocytic macrophage phenotype by promoting oxidative phosphorylation in macrophages and then delivering MSC-EVs to ameliorate lung injury in vivo [40]. Exosomes have also been reported to carry and present functional MHC-peptide complexes, which could modulate tumor-specific T cell activation [41].

As we known, exosomes are characterized by their cargoes of proteins and nucleic acids and free pass across various biological barrier, facilitating them as drug delivery vehicles like nanoparticles and liposomes. Nanoparticles emerge as a novel delivery of chemotherapeutic drugs in the treatment of cancers due to their attractive properties including biocompatibility, lower clearance rates, etc. [42]. However, the toxicology of nanoparticles in vivo still needs to be fully evaluated, especially after long-term exposure. In the contrast, MSC-EVs as naturally-occurring secreted EV from cells have advantages of becoming an ideal drug delivery vehicle. They are well tolerated and evade from host immune system. The target-homing ability of MSC-EVs further improve the efficacy [43].

Together, these results suggest that the immunosuppressive properties of MSC are mostly mediated by the release of EVs that convey regulatory molecules. Understanding the mechanisms underlying MSC-EVs functions is warranted to improve their utilization in therapy.

### The effects of MSC-EVs on the adaptive immune response

MSC-EVs can influence T and B lymphocytes and participate in the adaptive immune response. Mokarizadeh’s team initially demonstrated the effects of MSC-EVs on T cell proliferation. In the setting of experimental autoimmune encephalomyelitis, this group demonstrated that murine BM-MSC-EVs could express MSC-derived tolerogenic molecules, specifically PD-L1, galectin-1, and TGF-β1 and inhibit autoreactive lymphocyte proliferation, inducing secretion of the anti-inflammatory cytokines IL-10 and TGF-β. This result supports the view that MSC-EVs can induce tolerogenic signaling by promoting the generation of CD4 + CD25 + Foxp3+ regulatory T cells (Tregs) and apoptosis of activated T cells. Importantly, this group was the first to propose that tolerogenic and tolerogenic signaling molecules carried by MSC-EVs are key mediators involved in immunological tolerance [44]. Furthermore, Enrica Favaro’s team investigated whether hBM-MSC-EVs could suppress T cell activation in type 1 diabetes in vitro. This group found that coculture significantly increased the TGF-β, IL-10, IL-6, and PGE2 cytokine levels and skewed toward Foxp3+ regulatory T cells, suggesting a switch to anti-inflammatory T helper 2 (Th2) signaling in a type 1 diabetes model treated with hBM-MSC-EVs [45]. These findings were further corroborated by different independent in vitro studies that demonstrated that MSC-EVs decrease T-cell proliferation and activation due to the generation of Tregs and the immunosuppressive cytokine IL-22, showing similar potent immunoregulation as MSC [46–48].

The effects of MSC-EVs on the regulation of B lymphocytes have also been studied in in vitro experiments. Budoni et al. demonstrated that MSC exhibit strong inhibition of B cell proliferation and differentiation by inhibiting immunoglobulin secretion, and these effects could be fully reproduced by MSC-EVs in the presence of a CpG-stimulated PBMC coculture system in a dose-dependent fashion [48]. Interestingly, by using purified T, B, and NK cells or unfractionated PBMCs, a direct correlation between the degree of EVs mediating immunosuppression and uptake by immune effector cells demonstrate that B cells are mostly prone to incorporating EVs [49]. Together, these experiments support a pivotal and indispensible role for MSC-EVs in the adaptive immune response.

### The role of MSC-EVs in the innate immune response

**Fig. 1:**
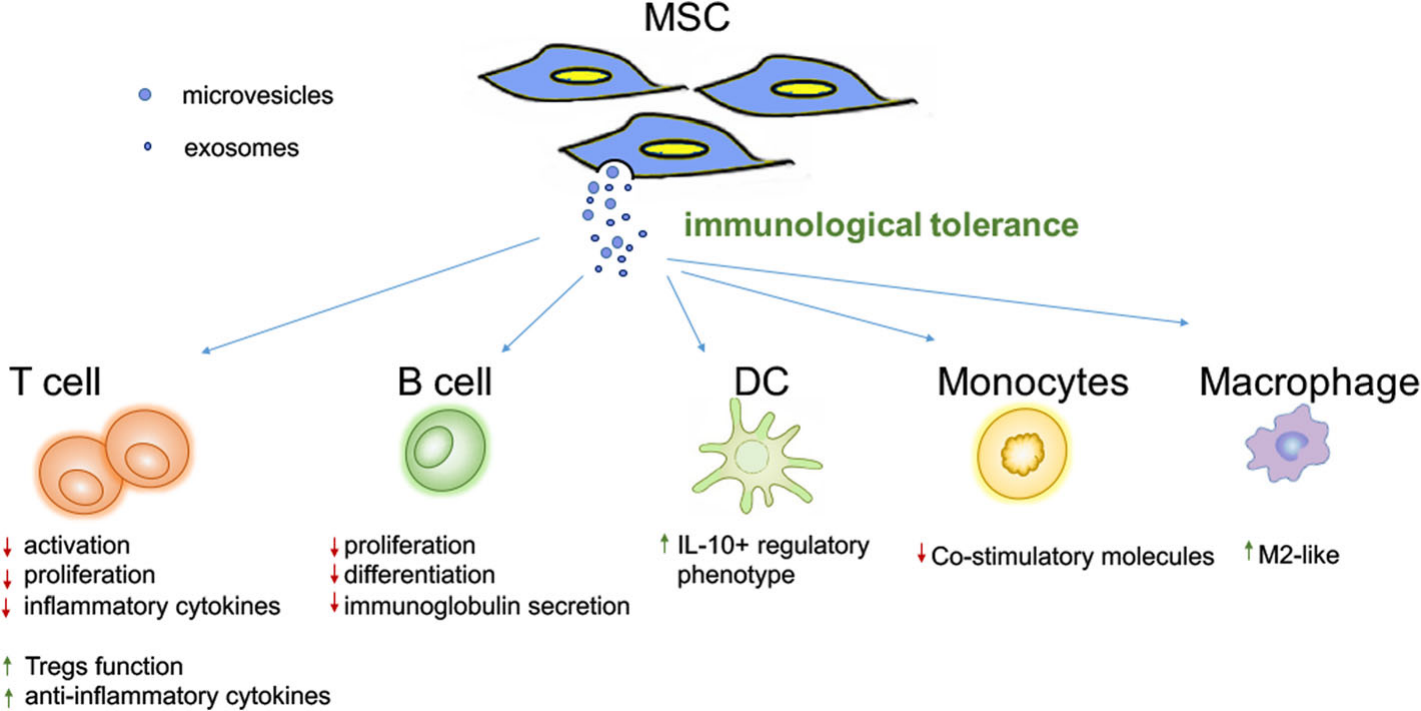
The role and mechanism of immunological tolerance of MSC-EVs on immune cells. The EVs derived from MSC play immunological tolerant role on the innate and adaptive immune responses including extensive immune cells. MSC-EVs could suppress the activation and proliferation of T cell and reduce production of inflammatory cytokines, while improve the Treg function and anti-inflammatory cytokines generation. Similarly, MSC-EVs play the suppressive role on the proliferation, differentiation, and immunoglobulin secretion of B cell. Considering the innate immune cells, MCS-EVs induce IL-10-expressing regulatory phenotype of DCs and inhibit the co-stimulatory molecules of monocytes. The macrophage would adopt anti-inflammatory M2 phenotype after MSC-EVs stimulation

In addition to their effects on cells involved in the adaptive immune response, MSC-EV suppressive functions have also been described for innate immune cells, such as dendritic cells (DCs), monocytes, and macrophages. For instance, Favaro et al. demonstrated that BM-MSC-EVs can skew monocyte-derived DCs toward an immature, IL-10-producing regulatory phenotype that associates with decreased levels of co-stimulatory molecules and increased IL-6, TGF-β, and PGE2 production in patients with type 1 diabetes [50]. Similar to DCs, a study has shown that MSC-EVs could polarize monocytes toward an M2-like phenotype although via the activation of TLR-dependent signaling in macrophages, which in turn induces CD4+ T cell skewing toward to Tregs [34, 51]. EVs from adipose-derived stem cells can transfer into macrophages to polarize the anti-inflammatory M2 phenotype via transactivation of arginase-1 by the carried, active STAT3 and then attenuate adipose inflammation and obesity [52]. In conclusion, these experiments demonstrate that MSC-EVs not only directly influence the immune state of adaptive immune cells but also indirectly change antigen presenting cell polarization into the anti-inflammatory state and induce the immunotolerance of T and B lymphocytes (Fig. 1).

### MSC-EVs as a potential therapy in various diseases

#### Autoimmune disease models

Most autoimmune disorders are described by a chronic inflammatory state; thus, improving the patient inflammation microenvironent becomes crucial. Recently, different immunotherapy methods, such as anti-cytokine therapies, signaling inhibitors, immune-modulating peptides, B-cell depletion, and apoptosis induction have been used for autoimmune diseases [53]. Although increasing survival rates and prolonging patient life spans, adverse and partial effects in a subgroup of patients have been identified. Therefore, to improve therapeutic effects and decrease side effects, the use of novel targeted immunotherapies based on MSC-EVs transfer RNA and protein for immunosuppression is attractive [51, 54].

There are accumulating studies that have examined the immunoregulatory function of MSC-EVs in autoimmune disease models. In an animal model of multiple sclerosis, it was shown that MSC-EVs contribute to inhibit auto-reactive lymphocyte proliferation and increase the secretion of anti-inflammatory cytokines, such as IL-10 and TGF-β, to reduce the effects of multiple sclerosis [44]. Similarly, in a Fas-deficient MRL/lpr mouse model of SLE, Liu’s team found that MSC-EVs were key for rescuing BM-MSC function after cell transplantation by transferring the Fas receptor in exosomes, which helped recipient cells reduce the intracellular miR-29b levels and ameliorate osteopenia [55]. Recently, in two autoimmune murine models of type 1 diabetes (T1D) and experimental autoimmune uveoretinitis (EAU), data have shown that MSC-EVs effectively prevent the onset of disease in T1D and EAU and inhibit activation of antigen-presenting cells and the development of Th1 and Th17 cells, which is as effective in modulating immune responses as MSC [56].

Osteoarthritis (OA) is a disease characterized by articular cartilage degradation, alteration in bone structure, synovial inflammation, and pain. Recently, a study has shown that MSC-EVs can reduce inflammatory arthritis by decreasing the plasmablast population and increasing the secretion of IL-10 Bregs in a collagen-induced arthritis (CIA) model [57]. In addition, a study reported that MSC-EVs can treat osteoarthritis when compared with synovium- or induced pluripotent stem cell (iPSC)-derived MSC-EVs. Data show that both were efficient in attenuating OA scores, but iPS-MSC-EVs had superior therapeutic effects on stimulating chondrocyte migration and proliferation in the CIA model [58]. In an animal model of synovitis, one group demonstrated a decrease in synovial lymphocytes together with downregulation of TNF-α transcripts in MSC-EV-treated joints, which may represent a promising therapeutic option for the treatment of synovitis [59]. These results raise the possibility that MSC-derived EVs may be an alternative to cell therapy for autoimmune disease prevention.

#### GVHD

The first landmark study highlighting the therapeutic potential of MSC-EVs has been published. In this study, MSC-EVs were administered to a patient with steroid-refractory acute GVHD failuire to conventional second-line treatment options [60]. First, the researchers tested the effects of polyethylene glycol (PEG)-based purifed MSC-EVs in a mixed lymphocyte reaction assay, and they observed that, in the presence of MSC-EVs, there were less immune cells in patient-derieved peripheral blood cells secreting proinflammatory cytokines. With these inspiring results, this group initiatied in vivo therapy. After undergoing hBM-MSC-EV treatment for four months in escalating doses, the patient’s clinical GVHD symptoms of cutaneous and mucosal manifestations were suppressed, and steroid administration was reduced, which was related to a reduction in the levels of the pro-inflammatory cytokines IL-1β, TNF-α, and IFN-γ and an increase in the levels of the anti-inflammatory molecules IL-10 and TGF-β in the PBMCs of the patients [60]. The study suggested that MSC-EVs can modulate the immune status of patients in a sustained manner. Subsequently, many animal models of GVHD were used to confirm the immunomodulatory mechanisms of MSC-EVs. Li Wang et al. sugested that hUC-MSC-EVs could also prevent aGVHD by modulating immune responses in a mouse model for allo-HSCT. Indeed, intravenously administrating hUC-MSC-EVs to recipient mice after allo-HSCT alleviates the manifestations of aGVHD by attenuating associated histological changes and significantly prolonging survival, which is related to reduced CD3 + CD8+ T cell numbers, reduced and IL-2, TNF-α, and IFN-γ serum levels, and higher IL-10 serum levels [61]. Lai P et al. suggested that MSC-exo could improve the survival of cGVHD mice by suppressing Th17 cells and inducing Treg [62]. It is imperative that more studies be performed to confirm the efficiency and elucidate the underlying mechanisms before MSC-EVs can be widely used in GVHD diseases.

#### Diabetes

In type 1 diabetes, islet transplantation might be the most efficient therapy [63, 64]. A suitable and effective method for overcoming the side effects of islet transplantation, including immune rejection and loss of islet function, is urgently needed. As a novel genetic material delivery vehicle, MSC-EVs have high delivery efficacy and will be a new way for improving the transplantation rate by suppressing the immune response. Indeed, Wen D et al. has shown that co-delivery of siFas and anti-miR-375 via hBM-MSC-EVs, which silences Fas and miR-375 in human islets, improves their viability and function against inflammatory cytokines in humanized NOD scid gamma (NSG) mice. Furthermore, intravenous injection of BM-MSC and PBMC co-cultured exosomes inhibited immune activity by inhibiting PBMC proliferation and strengthening Treg function [65]. Thus, gene overexpression or modified MSC-derived EVs might demonstrate stronger immune regulation than untreated MSC-derived EVs.

#### Inflammatory disease

The ability of MSC-EVs to regulate immune responses in inflammatory disease has also been studied. In kidney injury, accumulating activated immune cells can either repair or further aggravate injury in the kidney [66]. There are some clinical and preclinical study reports of the effects of MSC-EVs on kidney injury diseases (See Table 2). A single centre, randomized, placebo-controlled, phase II/III pilot study addressed the therapeutic impact of MSC-EVs in 40 patients with chronic kidney disease (CKD) [67]. Without demonstrating any MSC-EVs therapy related side effects in contrast with the control group during a one-year follow up, the MSC-EV-treated group significantly improved with regards to CKD symptoms as measured by the estimated glomerular filtration rate (eGFR), urinary albumin to creatinine ratio, blood urea, and serum creatinine levels compared with the control group. Taking immune factors into account, this study demonstrated that the anti-inflammatory cytokines TGF-β and IL-10 were significantly increased, and the pro-inflammatory cytokine TNF-α was decreased in MSC-EV-treated patients. Biopsies from these patients revealed the presence of activated CD133/Ki67 tubular cells compared with biopsies from control patients. This study suggested that the MSC-EV therapy is beneficial for balancing the immune environment and triggering regeneration in affected kidneys [67]. Previous reports have also highlighted the renoprotective effects of EVs during the early phase of AKI [68, 69]. In line with a study where UC-MSC-EVs protected kidneys from cisplatin-induced injury, another report noted that the protective effects of EVs against kidney ischemia-reperfusion (I/R) injury in the early phase are likely to be mediated through the innate immune system [46]. Furthermore, in an in vitro model of acute cisplatin injury, when exposed to BM-MSC-EVs and cord blood derived MSC-EVs (CB-MSC-EVs), kidney tubular cells lacking IL-10 expression acquired IL-10 mRNA, resulting in its translation and then rescue from injury, which suggests that horizontal mRNA transfer through EVs is a new mechanism for the MSC restoration ability [70].

**Table 2:** MSC-EVs application in clinical trial

Diseases	Clinical trial design	Potential mechanism	Refs
Graft-versus-host disease	individual treatment	decrease IL-1β, TNF-α and IFN-γ; increase IL-10 and TGF-β	[60]
Chronic kidney disease	phase II/III, single centre, randomized, placebo-controlled,	increase TGF-β and IL-10, decreaseTNF-α	[67]

A study hypothesized that hUC-MSC-EV-derived miR-181c could suppress the severe burn-induced inflammatory response in burned rats by downregulating the TLR4 signaling pathway and subsequently reducing NF-κB/p65 activation, which would prevent the release of pro-inflammatory factors such as IL-1β and TNF-α at the site of inflammation. The results demonstrated that exosomal miR-181c provides a potential target for the clinical therapy of patients with burn-induced inflammation [71]. Moreover, in a cutaneous wound model of diabetic rats, the application of LPS-preconditioned MSC-EVs relieves chronic inflammation and greatly enhances cutaneous wound healing overall by shuttling let-7b [34].

In the view that EVs can cross the blood-brain barrier, while MSC cannot, and deliver various therapeutic factors to the brain, MSC-EVs have been extensively tested as a useful treatment for the control of central nervous system (CNS) chronic inflammation [72, 73]. In an in vitro model for stimulation of the microglia cell line BV-2, a study has shown that mouse BM-MSC-EVs induce the immunosuppressive effects of microglia by modulating the activation of microglia involved in controlling MAPK phosphorylation, thereby leading to lower transcription of inflammation-associated genes, which represents an attractive souce for controlling CNS chronic inflammation [74].

Recently, an in vivo mouse model of pilocarpine-induced Status epilepticus, intranasal administration of hBM-MSC-EVs reached the hippocampus within six hours, protected the hippocampus from the loss of glutamatergic and GABAergic neurons, and greatly reduced inflammation [75]. Moreover, the neuroprotective and antiinflammatory effects were induced by decreasing the concentration of proinflammatory cytokines, increasing the expression of antiinflammatory cytokines and trophic factors, and reducing the occurrence of activated microglia [75]. Similarly, MSC-EV treatment significantly ameliorated inflammation-induced neuronal cellular degeneration, reducing microgliosis and preventing reactive astrogliosis and increasing the long-term outcome of a rodent model of inflammation-induced preterm brain injury [76]. These findings open a attractive way for novel cell-free-EV therapeutics to prevent chronic inflammation, particularly in the central nervous system.

### The advantages and discrepancies of MSC-EVs in immunoregulation

Current studies suggest that the application of MSC-EVs have most of the characteristic of MSC. Thus, the question becomes: why transplant MSC-EVs into the clinic if MSC have similar therapeutic effects? Because MSC-EVs have several advantages in clinical therapies compared with MSC, there is a push to use MSC-EVs in the clinic. First, because of their phospholipid bilayer, MSC-EVs can be easily prebanked, and they are more stable when freezing and thawing, which protects their contents from in vivo degradation, thus preventing problems associated with the rapid breakdown of soluble molecules. Most importantly, because they are equipped with a phospholipid bilayer, MSC-EVs cannot be altered by the inflammatory microenvironment, thus avoiding the polarizing effects of the immune environment as compared with MSC [77]. Second, as a nanostructure, MSC-EVs can easily travel through the pulmonary circulation or blood-brain barrier by intravenous injection and then freely circulate to reach further and deeper damaged sites to produce therapeutic effects [75]. Third, compared with cellular products, EVs do not self-replicate and thus lack endogenous tumor-formation potential. As self-replicating cells, although MSC therapy appears to be safe, is still has the risk of developing into sarcomas (soft tissue cancers derived from cells of mesenchymal origin). A study has shown that the application of other somatic stem cell entities, such as olfactory mucosal cells, could form tumors at the injection site after transplantation in patients with a spinal cord injury [78]. Fourth, as a cargo, MSC-EVs can be easily modified so that they are ideal delivery carriers by encasing molecules. In addition, owing to their small size and lower expression of membrane histocompatibility molecules, EVs have a lower possibility of immune rejection [79]. Moreover, EVs could be prepared in a uniform and standard manner in which their production procedures would be less complicated as more cell sources are available when immortalized MSC lines are used for manufacturing. Thus, EVs can be produced more easily in a scaled manner than cellular therapeutics. In view of these factors, using MSC-EVs as a therapeutic application is an attractive choice [80, 81].

There are still some remaining discrepancies that have been reported in the literature with regards to the different immunomodulatory mechanisms of MSC and MSC-EVs. In most experimental setups, MSC significantly inhibit the proliferation of activated T cells without inducing apoptosis. This effect has been reported to be mediated by the upregulation of intracellular pathways such as indoleamine 2,3-dioxygenase (IDO) [82, 83]. By contrast, no significant change in IDO activity was detected when treating with MSC-EVs [47, 48]. Although MSC-EVs have no effect on the CD3+ T cell production, similar to their parental MSC, MSC-EVs promote the proliferation of Tregs, enhancing the Treg/Teff ratio [47]. In addition, compared with parental MSC, secreted MSC-EVs are less able to skew the ratio of B cells and plasma cells in culture with PBMCs [66, 84]. MSC-EVs could retain the biological effects of parental MSC skewing monocyte-derived DCs toward an immature phenotype, but less effective than those modulating DC function [50]. Furthermore, Gouveia reported that MSC-EVs fail to suppress lymphocyte proliferation, suggesting that cell–cell contact may play a vital role in the immunosuppressive potential mediated by MSC [61, 68, 85]. The different and contradictory conclusions may stem from different experimental conditions and models, which suggest that when using MSC-EVs we need to consider ways to optimize the therapeutic outcomes of MSC-EVs.

### Optimization of the therapeutic outcomes of MSC-EVs

Many studies have reported that the biological features of MSC vary with individual donors, tissue source, culture conditions, and subpopulations. Heterogeneous MSC populations may explain the variable functions of MSC. To optimize MSC-EV therapy to be suitable for specific clinical indications, we should consider the following factors: (i) the source of the MSC, (ii) the composition of the culture media and growth factors used to culture the MSC, and (iii) the culture passages at which MSC are harvested and tested [86]. Indeed, before MSC-EV implication, it is imperative to devise a standardized approach for choosing the right MSC donor, MSC donor age, and source of MSC to obtain EVs with optimum immunosuppression functions. In addition, MSC-EVs have significant age-dependent differences in their immune profiles [87, 88]; thus, seeking a suitable source or population of MSC-derived EVs are warranted.

Emerging evidence demonstrates that MSC could be pre-stimulated by various cytokines to generate specific MSC-EVs, and after exposure to an inflammatory environment, such as high levels of interferon-gamma (IFN-γ) and tumor necrosis factor-alpha (TNF-α), MSC are provoked upon exposure to express iNOS (in rodents) or IDO (in humans), acquire immunosuppressive properties, and modulate lymphocyte activation [82, 89, 90]. Therefore, MSC pre-treated in vitro with immunosuppressive drugs or cytokines can enable them to enhance the expression and activity of IDO/INOS so that these types of MSC-derived EVs might better suppress immune reactions. For example, after MSC were treated with the cytokines IFN-γ and TNF-α, the resulting MSC-derived EVs signifcantly enhanced the expression of the immunomodulatory molecules [49]. In addition, a recent study analyzed the miRNA expression signature of IFN-γ-pretreated-MSC-derived EVs and found 62 significant differentially expressed miRNAs that are involved in immunoregulatory signaling pathways in these cells compared with control MSC-EVs, providing a better understanding of the immunosuppressive properties of the parent cells [91]. All of these data show that pre-treated MSC may enhance the immunoregulatory functions of MSC-EVs.

Exosomes and MVs may have different functional roles based on their different intracellular origins [92]. For this reason, it is necessary to determine the different composition and functional differences between EV subsets and whether their functions are prominent or specific to a given subtype (e.g., exosomes or MVs but not other EVs) [93]. Therefore, better knowledge of specific EV subtype markers is required for sorting. Recently, a new study characterized adipocyte-derived extracellular vesicle subtypes and identified distinct protein and lipid signatures for large and small extracellular vesicles, also underscoring the need to combine multiple approaches and markers to distinguish EV subpopulations because their specific composition may cause distinct metabolic responses in recipient cells and tissues [94]. Another new study revealed that some novel proteins are potentially useful when discriminating between MVs and exosomes. This study demonstrated that brain endothelial cell-released MVs were enriched in cytoskeletal and mitochondrial proteins, whereas exosomes were enriched in histones, and adhesion and ribosomal proteins. Phenotypic changes in donor cells induced by TNF were shown to lead to differentially expressed TNF signaling and immune response proteins in both MVs and exosomes [95]. In conclution, selecting certain subtypes of EVs using different EV markers or subpopulations of MSC that exhibit superior anti-inflammatory activity may optimize the therapeutic outcomes of MSC-EVs. In this sense, determining the total protein, lipids, or RNA content will call for the development of new methods [96, 97].

### Conclusion and prospects

Currently, there are several limiting factors for the clinical application of MSC-EVs: i) different labs use different methods to separate and purify EVs; thus, it is critical to find a highly effective and standard method for EV yields; ii) MSC-EVs comprise complex components, and further understanding of the mechanisms underlying MSC-EVs and finding specific components of EVs to efficiently obtain more stable and better therapeutic effects of MSC-EVs is required; and iii) the immunoregulatory functions of MSC-EVs have not been fully elucidated, and further exploration using in vitro and in vivo animal experiments remain to be performed as well as the toxicity and long-term safety in the human body needs to be further evaluated.

In sum, although the research on application of MSC-EVs still face many challenges, the advantages and potential of MSC-EVs are attracting increasing attention. As a non-cellular membrane structure, MSC-EVs have many advantages, including low immunogenicity, long half-life, in vivo stability, and high delivery efficiency. MSC-EVs are safer and more efficient than stem cell therapy, and they are one of the newer non-cellular biological therapies with great potential.

## Abbreviations

MSC: Mesenchymal stromal cells
EVs: Extracellular vesicles
MSC-EVs: MSC-derived extracellular vesicles
GVHD: Graft-versus-host disease
MS: Multiple sclerosis
SLE: Systemic lupus erythematosus
ISEV: International Society of Extracellular Vesicles
PEDF: Pigment epithelium-derived factor
Tregs: Regulatory T cells
DCs: Dendritic cells
T1D: Type 1 diabetes
EAU: Experimental autoimmune uveoretinitis
OA: Osteoarthritis
CIA: Collagen-induced arthritis
iPSC: Induced pluripotent stem cell
CKD: Chronic kidney disease
eGFR: Estimated glomerular filtration rate
CNS: Central nervous system
IDO: Indoleamine 2,3-dioxygenase
